# Mitigating physical hazards in food processing: Risk assessment and preventive strategies

**DOI:** 10.1002/fsn3.3727

**Published:** 2023-10-07

**Authors:** Helen Onyeaka, Dassalegn Daraje Jalata, Solomon Abate Mekonnen

**Affiliations:** ^1^ School of Chemical Engineering University of Birmingham Birmingham UK; ^2^ Department of Food Science and Nutrition Ethiopian Institute of Agricultural Research Addis Ababa Ethiopia

**Keywords:** food safety, physical hazards, quality control, regulatory compliance, risk mitigation

## Abstract

Physical contaminants in food, such as glass, metal, and plastic, can cause significant health risks and economic loss. This study explores these understudied physical hazards, aiming to provide comprehensive risk analysis and preventive solutions. Our research identified several key infiltration points in the food supply chain, including raw material sourcing and packaging stages. These hazards can be effectively mitigated by employing advanced technologies like metal detectors and optical sorting machines, along with stringent quality control measures. The findings offer valuable insights for stakeholders in the food industry, emphasizing the need for regulatory compliance and consumer education to ensure food safety.

## Graphical abstract

This article focuses on the risks associated with physical contaminants in food, such as glass, metal, and plastic. The study explores various infiltration points in the food supply chain and recommends the use of advanced technologies and quality control measures to mitigate these hazards. The findings highlight the importance of regulatory compliance and consumer education for ensuring food safety.

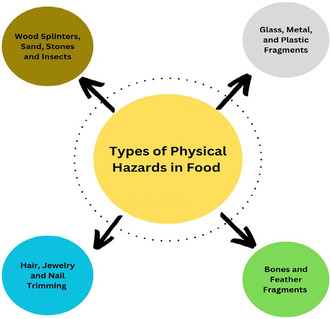

## 1 INTRODUCTION

Food safety is a critical aspect of the food industry, primarily focusing on microbial and chemical hazards (WHO, 2015). However, physical hazards in food can pose significant risks to consumer health and safety and have been a longstanding concern in the food industry (Drew & Clydesdale, 2015). The presence of physical contaminants, such as glass, metal, or plastic, in food products can lead to serious injuries or health hazards for consumers (Alamri et al., 2021). These hazards can occur at various food production and supply chain stages, from raw material sourcing to processing, packaging, and distribution (Mahalik, 2014; Pettoello‐Mantovani et al., 2021). They pose risks to consumers, and understanding the risks associated with physical hazards in food is crucial for ensuring the safety of consumers and maintaining the food industry's integrity.

Consuming food with physical hazards can result in anything from minor abrasions or cuts to more serious issues like choking, damage to the internal organs, or even death. In addition to the adverse health effects, cases from physical hazards in food can potentially cause food manufacturing companies to be in considerable financial losses, damages to their reputations, and perhaps legal repercussions. As a result, it is crucial for all parties involved in the food sector to be aware of the risks posed by physical hazards and take preventative measures to reduce them.

One primary contributing factor of physical hazards in food is the failure to effectively apply quality control and safety measures across the manufacturing and distribution phases. Physical contaminants can unintentionally infiltrate the food supply chain through different channels, such as equipment breakdowns, incorrect handling, or foreign items in raw materials (Moerman, 2017). A comprehensive strategy is, therefore, needed to identify possible sources of physical contaminants and put preventive measures in place to reduce their occurrence.

Recent technologies for identifying and removing physical hazards throughout the production and packaging processes include metal detectors, X‐ray systems, and optical sorting machines (Nuutinen, 2023). The risk of contamination is reduced by these technologies' ability to identify the most minute foreign materials. To preserve the integrity of food items and protect consumer health, stringent adherence to quality control standards, routine inspections, and thorough testing processes are very important.

As a preventive measure, regulatory authorities play a crucial role in the food industry by establishing guidelines and standards (Kotsanopoulos & Arvanitoyannis, 2017). Governments and international organizations set the allowed levels of physical hazards in food, which also specify the duties of food producers and processors in maintaining compliance (Mortimore & Wallace, 2013). However, the effectiveness of these laws rests on their application and the food industry's readiness to embrace best voluntary practices. Additionally, educating consumers on the dangers posed by physical hazards might enable people to make informed decisions and to demand accountability from food manufacturers.

This article aims to comprehensively understand physical hazards in food, their sources, potential consequences, and strategies to minimize their occurrence.

## 2 WHAT ARE PHYSICAL HAZARDS IN FOOD?

A physical hazard is any foreign matter presented in food or a naturally occurring object that can cause illness or harm to the consumer (Cavalheiro et al., 2020). Unsanitary conditions during food production, processing, storage, and distribution are associated with foreign materials. These materials are hazardous due to their sharpness, hardness, size, or shape, which may cause lacerations, perforations, wounds, or choking hazards (Bujang et al., 2020; Kenner, 2001). Fragments of glass, which can occur during handling, transportation, or improper storage of food and beverages in glass containers; metal, such as broken parts, screws, or wire bristles that can be incorporated during processing; stone or pieces of rock enter the food during harvesting or processing of agricultural products, wood coming from pallets, crates, or utensils used in food processing or storage, plastic resulted from damaged or degraded packaging materials or equipment components, bone due to inadequate deboning or processing practices of meat and fish products, hair or fibers and jewelry that occur during food handling, and insects that arises due to improper pest control measures are some common types of physical hazards in food (Cavalheiro et al., 2020; Rhodehamel, 1992).

**FIGURE 1:**
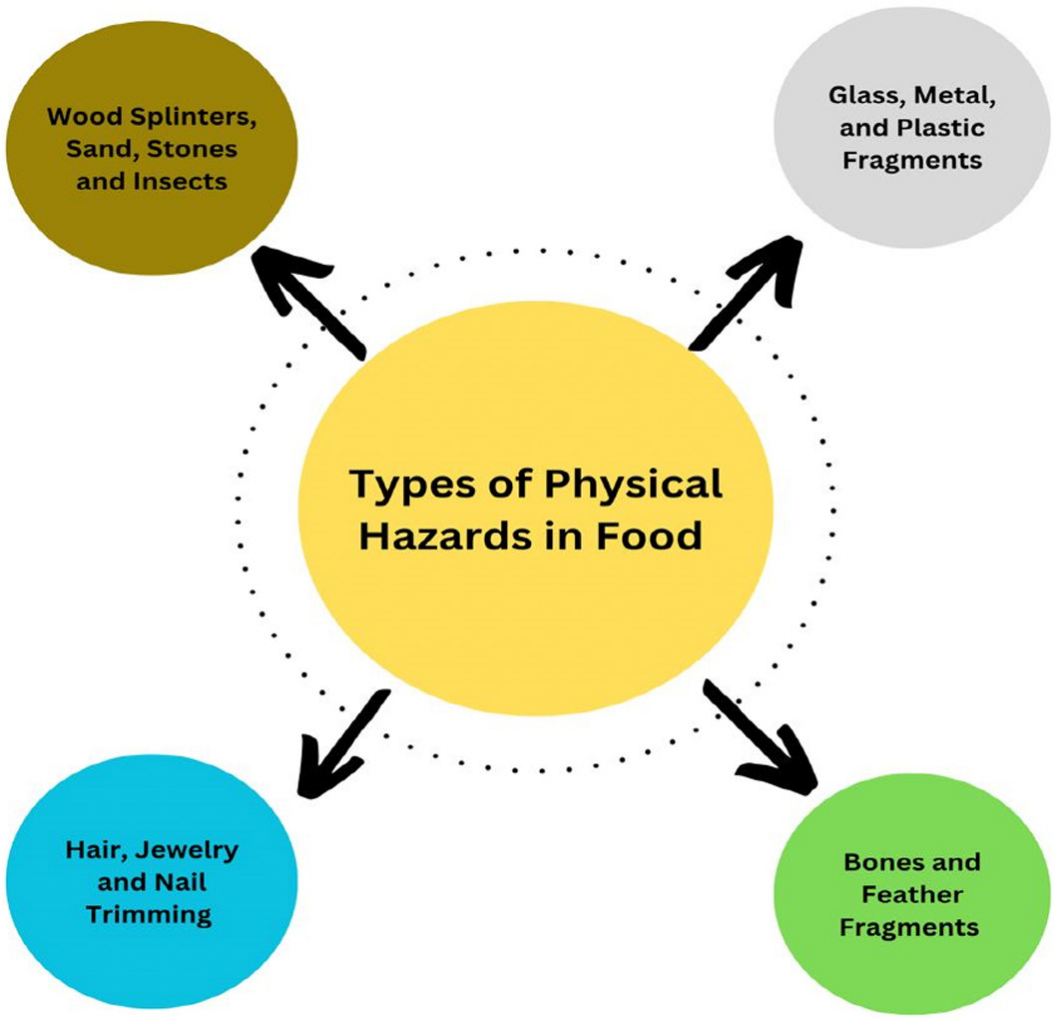
Types of physical hazards in foods.

Foreign materials can be classified into two categories: those that are unavoidable and those that can be avoided. Small, incidental, inherent, or unintentionally added foreign materials that are present in the food product despite all necessary quality assurance methods being followed and that cause minimal risk to consumers are considered unavoidable materials. Common foreign materials such as dirt on potatoes, remnants of insect fragments in figs, and stems in blueberries fall under this category. On the other hand, some foreign materials can be avoided by using proper methods. Small glass fragments, pieces of jewelry, animal debris, and pieces of plastic are among the avoidable physical hazards that can be present in food (Singh et al., 2019). Figure 1 shows the types of physical hazards in foods.

### 2.1 Sources and detection of physical hazards

Physical hazards can be introduced unintentionally into food during the process of harvesting, processing, manufacturing, packaging, handling, or transportation, from “gate to plate” or “farm to fork”, or due to food tampering or intentional sabotage (Nkosi et al., 2022). However, naturally occurring physical hazards may be present in raw materials if not properly removed during harvesting or processing (Pettoello‐Mantovani et al., 2021). Sand, sticks, and leaves can appear in the product together with the raw materials, as can fragments of bones in meat and fish and seeds and fruit stones in fruits (Szumicka, 2022). Foreign materials such as metal and plastic fragments may be transferred to the products during processing, packaging, or transportation (Banach et al., 2017; van Asselt et al., 2017). Due to the failure of workers to observe sanitary standards and to apply the correct protective clothes, materials such as jewelry, hair, and buttons can be present in food (Asselt et al., 2016). Also, due to not observing the principles of good manufacturing practice, glass, pieces of gypsum, or paint can be present in food (Szumicka, 2022). Table 1 shows the sources of physical hazards in foods.

**TABLE 1:** Sources of physical hazards in foods.

Food	Hazard	Source of hazards	References
Meat and meat products	Bones Animal hair Metal fragments, hard plastic, glass, and wood	Raw materials Processing and packaging	Cavalheiro et al. (2020), Das et al. (2019), and Park et al. (2017)
	Insects	Processing and Packaging	
	Hair, jewelry, and nail trimmings	Handling and transportation	
Wild meat	Bullet particles and bone fragments	Raw materials	Nkosi et al. (2022)
Chicken meat	Bone and feather fragments	Raw materials	Banach et al. (2017) and Cavalheiro et al. (2020)
Pork meat	Needle and metal fragments	Raw materials	Coelho et al. (2019)
Milk and milk products	Paper, plastic, glass, metal, and rubber seals	Processing and packaging	Aguiar et al. (2018), Asselt et al. (2016), and van Asselt et al. (2017)
	Hair, jewelry, soil/sand, and insect	Handling and transportation	
Egg products	Eggshell fragments	Processing	van der Fels‐Klerx et al. (2016)
Fish and fish products	Fish bone fragments	Raw materials	Anggraeni et al. (2021) and Park et al. (2017)
	Hard plastic, metal, glass, and wood splinters	Processing and packaging	
	Hair and insect	Handling and transportation	
Dried mango	Wood splinters and grains of sand	Raw materials	Tarnagda et al. (2021)
	Metal and boxes	Processing and packaging	
	Hair and nail	Handling and transportation	
Melon snack	Sand/soil, melon seed, and metal fragments	Raw materials	Sobukola et al. (2008)
	Rodent hair	Handling and transportation	
Seaweed food	Plastic fragments	Raw materials	Banach et al. (2020)
Infant food	Stones	Raw materials	Pettoello‐Mantovani et al. (2021)
	Glass, metal, and plastic fragments	Processing and packaging	
	Wood splinters	Handling and transportation	
Rice and other grain‐based foods	Unwanted grains and sticks	Harvesting and processing	Bühler (2014)
	Glass, metal, and insects	Processing and packaging	
Fresh fruits and vegetables	Dust, sand, wood, and metal pieces	Processing	Hussain and Gooneratne (2017)

Foreign materials can be detected in food through mechanical, optical, and electromagnetic interaction techniques. Mechanical techniques detect foreign bodies by means of size or weight differences between the foreign body and the food product and use simple techniques like sieving, filtering, and centrifuging (Aladjadjiyan, 2006). Optical techniques find foreign materials by means of shape and/or color analysis through the interaction of light (the visible part of the electromagnetic spectrum) with food products and foreign materials (Aladjadjiyan, 2006; Mohd Khairi et al., 2018). In addition, foreign materials can be detected through electromagnetic interaction by using different parts of the electromagnetic radiation spectrum, such as X‐ray inspection, microwave inspection, near‐infrared inspection, ultraviolet inspection, nuclear magnetic resonance, magnetic field system, electrostatic techniques, and ultrasonic techniques (Demaurex & Sallé, 2014; Nuutinen, 2023). Table 2 shows methods for detecting physical hazards in food.

**TABLE 2:** Techniques for detecting physical hazards in food (Aladjadjiyan, 2006).

Technique	Wavelength	Food product	Foreign bodies
Magnetic	Not applicable	Loose and packaged food	Metals
Microwave	1–100 mm	Fruits, possibly others containing water	Fruit pits
Nuclear magnetic resonance	1–10 mm + magnetic field	Fruits and vegetables	Fruit pits and stones
Infrared	700 nm − 1 mm	Nuts, fruits, vegetables	Nutshells, stones, pits
Optical	400–700 nm	Any loose product, fruits, vegetables	Stones, stalks
Ultraviolet	1–400 nm	Meat, vegetables, fruits	Fat, sinews, stones, pits
X‐rays	<1 nm	All loose and packaged goods	Stone, metal, rubber, glass, plastic, bone
Ultrasonic	Not applicable	Potatoes in water	Stones

### 2.2 Consequences of physical hazards and their risk levels

Physical hazards present in food that can pose a potential risk to consumers depend on several factors. These factors include the size of the foreign material found in the food product, the type of contaminated food, the physical characteristics of the foreign material, and the consumer who is eating the food product (Nkosi et al., 2022; Table 3). While the effects of physical hazards may vary from case to case, ingesting foreign objects can result in injuries such as cuts, lacerations, internal damage, choking, or posing immediate health risks (Aladjadjiyan, 2006; Das et al., 2019). Furthermore, it can also lead to secondary infections of the mouth, tongue, gums, teeth, esophagus, and other digestive system organs (Aguiar et al., 2018; Kenner, 2001). Notably, foreign material such as hair, insects, or sand found in food products is unlikely to cause serious injuries to an exposed individual (Anggraeni et al., 2021; Singh et al., 2019). Table 4 shows the potential injuries for the main physical hazards.

**TABLE 3:** Level of physical hazards injury (Singh et al., 2019).

Food	Size of physical hazard	Injury level
Infant foods	Any size (including small particle <2 mm)	High
Beverage	2 mm or larger in size in any one dimension	High
All other foods (except infant food and beverage)	2 mm or larger in size in any one dimension	Moderate
All other foods (except infant food)	2 mm in size in all dimensions	Low

**TABLE 4:** Potential injuries of main physical hazards.

Material	Potential injuries	References
Glass	Cuts to tongue and mouth, gastrointestinal perforations and injuries	Aladjadjiyan (2006), Olsen (1998), and Rhodehamel (1992)
Metal	Cuts to tongue and mouth, oral and throat laceration, pose infections	Aguiar et al. (2018), Aladjadjiyan (2006), and Rhodehamel (1992)
Plastic	Choking to the throat, intestinal damage, pose to infections	Aladjadjiyan (2006), Molina and Benedé (2022), and Rhodehamel (1992)
Bones	Choking to the esophagus, trauma, perforation, and wounding intestine	Aladjadjiyan (2006), Gunn (1966), and Olsen (1998)
Stones	Choking to the throat, broken teeth	Aladjadjiyan (2006) and Rhodehamel (1992)
Wood	Cuts to mouth and tongue, choking to the throat, pose to infections	Aladjadjiyan (2006) and Rhodehamel (1992)
Insects and other pests	Foodborne illness, trauma, choking to throat	Aguiar et al. (2018), Aladjadjiyan (2006), and Cavalheiro et al. (2020)
Personal effects (hair, jewelry)	Choking to the throat, tooth breakage	Aladjadjiyan (2006) and Rhodehamel (1992)
Allergenic materials (nuts, shellfish, gluten‐containing particles)	Minor to severe allergic reactions to individuals with allergies or sensitivities	Shridhar and Sharma (2009)
Corn chip	Laceration to esophagus	Meislin and Kobernick (1983)

Ingesting foreign materials may increase trauma for at‐risk populations such as children, the elderly, the visually impaired, and postoperative patients (Aguiar et al., 2018). Evidence suggests that 1%–5% of the foreign objects ingested (swallowed) by people result in minor to serious injury (Aguiar et al., 2018; Aladjadjiyan, 2006). Most ingested foreign objects (80%–90%) pass through the gastrointestinal tract spontaneously, with the remainder requiring removal either by endoscopy or, less frequently, surgery. For sharp objects, the risk of tissue perforation in the gastrointestinal tract is greater and requires greater surgical removal than other kinds of ingested foreign materials (Aladjadjiyan, 2006).

According to the FDA (Food and Drug Administration), mouth or throat injuries in the form of lacerations and minor dental damages are the most frequent type of injuries (Marshall et al., 2016). On the other hand, less frequent injuries are reported on gastrointestinal distress (nausea, vomiting, and diarrhea), choking, headache, dizziness, puffy face, rash, fever, chest pain, lost voice, nosebleed, collapsed lung, seizure, pain in the arm and shoulders (Olsen, 1998).

## 3 IMPORTANCE OF PREVENTIVE MEASURES

Providing safe, wholesome, and nutritious food to consumers requires the participation of every individual. To accomplish this, every party involved in the food chain is responsible for every step of the processing, from the farm or food production stage through processing, storage, distribution, retail sale, and consumption. As a result, preventing physical hazards in food is not solely the responsibility of one group but rather an obligation of collective participation (Marshall et al., 2016). Preventing physical hazards in food is a matter of safety and ethical responsibility toward consumers. When considering the prevention of physical hazards in food, legislation, and regulation have to indicate or concern the properties of food products, including their raw material, processing and packaging, handling and transportation, labeling, precautions and instructions for their use, and any guidelines relating to food products available to consumers (Szumicka, 2022). Moreover, food processors, food businesses, and food‐service operators must adhere to strict regulations to prevent physical hazards and avoid legal consequences such as product recalls, fines, or closure (Marshall et al., 2016). They also have the responsibility of prioritizing consumers' welfare and safety, which involves ensuring that the food they produce is free from physical hazards that could potentially harm consumers; they need to be transparent in providing accurate labeling and information about potential allergens or foreign objects that may be present in their product. Establishment of a quality control and monitoring system, provision of continuous training to employees and establishment of clear accountability, seating strong relationship between food producers and processors in a bid to get high‐quality ingredients that meet safety standards, development and implementation of continuous assessment and update system of food processing to mitigate risks associated with physical hazards, and establishment of a responsive system that promptly address the incident of physical injuries by taking corrective actions and communicate transparently with consumers are an important consideration to prevent foods from being contaminated by physical hazards.

Food products that do not meet safe levels of physical hazards can damage consumer trust and harm a company's reputation, leading to decreased sales and a long‐term negative impact (Pettoello‐Mantovani & Olivieri, 2022). The application of legislation on food product safety varies from region to region or state to state (Pettoello‐Mantovani & Olivieri, 2022). The Food and Agriculture Organization and the World Health Organization have set food standards on an international scale that cover the safety of food from field to table, complying with legal requirements. However, acceptance of these standards is voluntary and depends on the interest of individual governments in implementing them (Marshall et al., 2016; Pettoello‐Mantovani et al., 2022). For instance, the FDA has established a maximum level of physical hazards in food that pose no significant risks to human health, including tiny insect parts and dirt (Aladjadjiyan, 2006).

In contrast, no particular legislation or limits exist for physical hazards such as contaminants in food in Europe (Banach et al., 2020). This indicates that understanding the risks of physical hazards is not the same among regulatory bodies. Thus, focusing on preventing and controlling physical hazards in food is essential to ensuring consumer safety.

## 4 STRATEGIES TO MINIMIZE PHYSICAL HAZARDS

### 4.1 Supplier control and auditing

To ensure food safety and reduce physical risks, the food sector must strictly supervise and audit its suppliers (Kotsanopoulos & Arvanitoyannis, 2017). Establishing rigorous quality control standards for suppliers, including inspection of raw materials for physical hazards (Okpala & Korzeniowska, 2021), effective implementation includes assessing and choosing suppliers based on their compliance with safety regulations, outlining expectations for hazard control, conducting routine audits, and implementing remedial measures as needed (Motarjemi & Warren, 2023). Maintaining a solid supplier relationship and consistently enhancing safety procedures require ongoing communication, performance monitoring, collaboration, and training. Processes must be regularly reviewed and updated to meet new physical hazard issues and incorporate new knowledge.

### 4.2 Facility design and maintenance

Designing food processing facilities with materials resistant to physical hazards and conducting regular equipment maintenance and inspections are critical for ensuring food processing operations' safety and efficiency (Kutz, 2019).

### 4.3 Good manufacturing practices

Good manufacturing practices (GMP) is crucial for the food sector to reduce physical risks. GMP covers policies and processes focusing on supplier control, cleaning and sanitation, documentation and record‐keeping, equipment maintenance, employee hygiene and training, and facility design (De Oliveira et al., 2016). Businesses can significantly lower the risk of physical hazards in food production by designing facilities with hazard prevention in mind, maintaining equipment properly, training employees on proper food handling and hygiene practices and ensuring personnel follow hygiene protocols, implementing supplier control measures, practicing effective cleaning and sanitation, and maintaining accurate records (Schoenfuss & Lillemo, 2014). GMP adoption not only promotes consumer confidence and maintains regulatory compliance, but it also guarantees food safety and quality.

### 4.4 Effective quality control

Implementing robust quality control procedures, including visual inspections, sieving, filtering, or metal detection systems.

### 4.5 Traceability and recall systems

Strong traceability systems that follow products from the raw materials to the final distribution are necessary for effective quality control and mitigating food safety problems (Agrawal et al., 2018; Onyeaka et al., 2021). Maintaining accurate records and implementing traceability solutions allow for the quick identification, isolation, and recall of impacted products in the case of physical hazard incidents. By doing this, the effect on consumers is lessened, and physical hazards are kept from spreading throughout the market.

### 4.6 Education and awareness

To maximize physical safety in the food business and strengthen quality control efforts, comprehensive training and education programs for staff are crucial (Mortimore & Wallace, 2013). These initiatives make sure that employees fully comprehend the relevance of identifying and avoiding physical hazards. Employees receive instructions on proper handling procedures, how to operate the equipment, and identifying any potential physical hazards that could arise through the production process. Businesses may reduce risks and maintain a safe working environment by providing staff with the appropriate information and skills (De Oliveira et al., 2016). This improves the overall quality control methods used in the production process.

Increasing awareness and implementing these strategies can enhance food safety and protect consumers from potential harm.

## 5 CONCLUSION

Physical hazards refer to foreign objects unintentionally or in some cases as part of sabotage present in food that can cause harm or injury if consumed, such as glass, metal, stones, wood, plastic, bone fragments, insects, or other foreign materials. The sources of physical hazards can be traced to raw materials, processing and packaging, and handling and transportation. Physical hazards in food can have severe consequences for consumer health, legal compliance, and brand reputation. By understanding the sources, and potential risks and implementing preventive measures, food businesses can minimize the occurrence of physical hazards and ensure the safety of their products. Prioritizing food safety through supplier control, facility maintenance, GMP, quality control, traceability systems, and continuous education is essential to protect consumers and maintain trust in the food industry. It is, therefore, very important to understand physical hazards, implement preventive measures, and continuous improvement to assure food safety and protect consumers.

To ensure food safety and safeguard consumers, the industry must comprehensively understand physical hazards, implement proactive preventive measures, and continuously strive for improvement. This requires a collaborative effort among all stakeholders involved in the food supply chain, from producers to distributors, retailers, and regulatory bodies. By publishing research, sharing best practices, and promoting knowledge exchange in this area, we can further enhance food safety practices and protect consumers from the potential harm caused by physical hazards. Continued efforts in understanding, preventing, and addressing physical hazards will contribute to a safer and more trustworthy food industry, fostering consumer confidence and satisfaction.

## AUTHOR CONTRIBUTIONS

**Helen Onyeaka:** Conceptualization (equal); writing – original draft (equal); writing – review and editing (lead). **Dassalegn Daraje Jalata:** Writing – original draft (equal); writing – review and editing (equal). **Solomon Abate Mekonnen:** Writing – original draft (equal); writing – review and editing (equal).

## FUNDING INFORMATION

The authors declare that no funds, grants, or other support were received during the preparation of this manuscript.

## CONFLICT OF INTEREST STATEMENT

The authors declare that they have no known competing financial interests or personal relationships.

## Data Availability

The data that support the findings of this study are available from the corresponding author upon reasonable request.
